# A review and comparative analysis of the risk-needs-responsivity, good lives, and recovery models in forensic psychiatric treatment

**DOI:** 10.3389/fpsyt.2022.988905

**Published:** 2022-10-31

**Authors:** Maximilian Lutz, Davide Zani, Michael Fritz, Manuela Dudeck, Irina Franke

**Affiliations:** ^1^Department of Forensic Psychiatry and Psychotherapy, University of Ulm, Ulm, Germany; ^2^Department of Forensic Psychiatry, Psychiatric Services Grisons, Chur, Switzerland

**Keywords:** recovery, mental disorder, offending, risk, treatment, GLM, RNR

## Abstract

Forensic mental health care primarily focuses on aspects of safety. Treatment is involuntary, and personal rights are highly restricted. Both direct and indirect coercion and significant power imbalances can impede not only the psychological state of inpatients but also their treatment motivation and the therapeutic process in general. However, successful treatment is essential to enable patients to regain their freedom. Therefore, the question arises whether and how health professionals, without disregarding the potential risks, can enable forensic psychiatric patients to experience meaningfulness and self-efficacy in their lives. In offender rehabilitation, the Risk-Need-Responsivity (RNR) model and Good Lives Model (GLM) are widely established theories. The RNR model focuses not only on the risk of recidivism but also on those needs of a person that provoke or prevent criminal behavior and the individual’s ability to respond to various kinds of interventions. In contrast, the GLM aims to reduce the risk of re-offending by enabling an individual to live a “good life,” i.e., a meaningful and fulfilling life. Originally developed in correctional services, i.e., for offenders without severe mental disorders, both the RNR model and the GLM have also been tested in forensic psychiatric treatment contexts. The Recovery Model is based on the concept of personal recovery in mental health care and is understood as the development of a sense of purpose and mastery in one’s own life during the process of coping with the sequelae of a mental disorder. It is a central element of rehabilitation in general, but is also being increasingly applied in forensic psychiatric treatment settings. This review aims to compare the central concepts of the three models, in particular regarding personal development, and the current evidence for their efficacy in mentally disordered offenders.

## Introduction

The past history of psychiatric care is notoriously characterized by taking control of the lives of people with mental illness and segregating them in environments away from society (1). Until the twentieth century, the only treatment method deemed credible was what we now call *institutionalization* [or *total institutionalization*, according to Goffman (2)]. The standard treatment was to isolate mentally ill people in institutions (asylums), where every area of life was tightly planned, imposed from above and performed simultaneously and stereotypically together with all the inhabitants of these facilities. Early rehabilitation procedures were far from being scientific and made little distinction between the individuals’ diagnoses. In the early 1950s, with the discovery of the first antipsychotic, chlorpromazine, and its implementation into clinical practice (3), the perceptions of the scientific community and society as to how individuals with psychiatric pathology should be treated slowly began to change. It is precisely because of pharmacological and clinical advances that we saw the emergence of a series of movements that shaped contemporary psychiatry. The first major movement was the normalization movement in the 1970s (4, 5), which highlighted the ethical need to integrate people with any kind of disability back into society, followed by the deinstitutionalization in the 1980s (6), that aimed to transfer mental health care from institutions back to families and communities, and lastly the pioneering work of Deegan (7) and Anthony (8) and the Consumer/Survivor movement in more recent times (9), which insisted on ending the idea of unmodifiable mental illness and disability by introducing the concepts of recovery and rehabilitation into clinical practice. Today, this strand of thought takes the name *recovery movement*.

Because forensic psychiatry has a different mandate than general psychiatry and still often resembles the concept of a total institution, the movements mentioned above did not become established in forensic psychiatry at the same speed and to the same extent as in general psychiatry. Admission to treatment in forensic psychiatry usually is involuntary. In contrast to other psychiatric and psychotherapeutic settings, intrinsic insight and motivation to change are often not given—at least in early stages of therapy—and individual treatment goals might collide with or be subordinated to public safety interests. Furthermore, because of the mandatory character of treatment and the dual role of the professional (i.e., therapist and risk manager), some authors have questioned whether the essential principles of psychotherapy, particularly voluntariness and confidentiality are given (10). However, the patient’s decision on whether to participate in therapy (assuming that decision-making capacity is given) and the reduction of risk factors, such as specific symptoms of the mental disorder, are crucial for risk assessment and further decisions on release. Rates of re-offending are the primary measure in evaluating forensic psychiatric treatments, and this criterion is mainly directed toward public safety interests and more or less neglects the individual patient’s perspective. Therefore, legally mandated treatment of mental and substance use disorders might be considered as counterintuitive to some of the core assumptions of psychotherapy. Nevertheless, mandated treatment has proven to be effective in terms of reducing re-offending in individuals with a severe mental disorder, although other outcome measures, such as readmission rates and mortality, have not necessarily (11).

As a further consequence of safety principles, forensic psychiatric facilities, according to Tomlin et al., are characterized by different forms of direct and indirect power imbalances and coercion across individual (i.e., relational, tangible), institutional (i.e., built environment, activities, culture, atmosphere, therapeutic aspects, security, practicality), and systemic (i.e., regulatory, temporal) levels; the amount of perceived restrictiveness depends on whether the approach to care is more caring (vs. custodial) and whether the resident is perceived as risky (12). The authors further argued that, because of the negative outcomes of restrictive measures, it is necessary to reflect critically on practices, procedures, and policies in forensic care settings (12). For example, recent studies indicated a negative effect of highly restrictive treatment settings on the quality of life (QoL) of forensic psychiatric inpatients, especially regarding symptoms of depression and suicidality (13, 14).

Given the above-mentioned characteristics of secure treatment settings and their effects on inpatients, on the one hand the question arises whether and how forensic psychiatric and psychotherapeutic treatment can hinder or promote personal development, in particular coping with severe mental illness, and whether such an effect also influences the risk of re-offending. On the other hand, assuming that it is methodologically rather difficult to prove a causal association between rather abstract concepts (such as QoL) and reoffending, in the treatment of mentally disordered offenders (MDOs) one might consider whether quantitative outcome measures such as the rates of reoffending should be seen as being independent from qualitative ones, in particular compliance with ethical and humanitarian principles. This review aims to compare three prominent models applied in the rehabilitation of MDOs, the Risk-Needs-Responsivity (RNR) model, Good Lives Model (GLM), and Recovery Model (RM), and to discuss their strengths and limitations.

## Rehabilitation models applied in forensic psychiatry

### Risk-needs-responsivity model

The RNR model is a prominent model of offender rehabilitation that was developed in Canada on the basis of the Psychology of Criminal Conduct and General Personality and Cognitive Social Learning Model (GPCSL) (15). Treatments that followed the RNR concepts were able to reduce sexual recidivism (16), violent recidivism (17), and general recidivism (18). According to the RNR rationale, criminal behavior develops on the basis of personality predisposition and learning and is influenced by the individual’s expectations and the consequences of criminal behavior. The model guides therapy by focusing on the risk of re-offending without disregarding each patient’s individual characteristics. The treatment of offenders should proceed according to the three core principles of risk, needs, and responsivity described below.

The principle of *risk* addresses the question “Who should receive treatment?” The amount of treatment delivered to the offender should match their risk of re-offending, i.e., offenders with high-risk profiles should receive more intense treatment and management. Risk can be addressed with different evidence-based instruments, so the forensic psychotherapist must have knowledge of current risk assessment procedures and risk factors.

The principle of *needs* focusses on “What should be treated?” Treatment and management should focus on the identified criminogenic needs (i.e., dynamic risk factors empirically associated with recidivism). The central risk/need factors (the *Big Eight*) are as follows: (1) history of antisocial behavior, (2) antisocial personality pattern, (3) antisocial/procriminal attitudes, (4) antisocial associates, (5) problematic circumstances of home (family/marital relationships), (6) problematic circumstances at school/work, (7) few/no prosocial recreational activities, and (8) substance abuse (15). The 4 minor needs, i.e., self-esteem, vague feelings of personal distress, major mental disorder (e.g., schizophrenia or bipolar disorder), and physical health, are considered as non-criminogenic needs and should only be focused on in treatment if relevant for aspects of treatment responsivity.

The principle of *responsivity*, which consists of general and specific responsivity, answers the question “What should treatment look like?” Services should be delivered in a way that maximizes their effectiveness, i.e., facilitates the offender’s ability to learn from the program. The Responsivity aspect focuses on skills acquisition and enhancement and appropriate reinforcement and disapproval; furthermore, the style of delivery should address offenders in a way that matches their learning skills, motivation, abilities, and strengths. *General responsivity* factors are aimed at influencing behavior and should be based on established cognitive social learning methods, and *specific responsivity* factors are described as “personal characteristics that regulate an individual’s. ability and motivation to learn” (19), including mental health problems.

### Good lives model

The GLM of offender rehabilitation is based on the ethics of human dignity and agency (20) and was originally developed for sex offender treatment. Its core assumption is that all individuals have similar aspirations and needs in life and that they formulate and choose goals, make plans and act feely in order to achieve them. Criminality is considered as a maladaptive strategy for meeting life values or as a failure in pursuing relevant life goals by prosocial means (21). In accordance with that rationale, treatment interventions should enable offenders to achieve personally meaningful goals or to develop the necessary knowledge, skills, and opportunities to be able to satisfy their life values without delinquent behavior.

The GLM describes 11 classes of primary goods: (1) life (including healthy living and functioning), (2) knowledge (how well informed one feels about things that one considers important), (3) excellence in play (hobbies and recreational pursuits), (4) excellence in work (including mastery experiences), (5) excellence in agency (autonomy and self-directedness), (6) inner peace (freedom from emotional turmoil and stress), (7) relatedness (including intimate, romantic, and familial relationships), (8) community (connection to wider social groups), (9) spirituality (in the broad sense of finding meaning and purpose in life), (10) pleasure (the state of happiness or feeling good in the here and now), and (11) creativity (expressing oneself through alternative forms). Secondary (or instrumental) goods are approach goals that serve as means to secure primary goods (22). The pathway to offending is either direct (i.e., offending behavior is explicitly or implicitly chosen to achieve primary goods) or indirect (i.e., failures or disappointments in the pursuit of a primary good and maladaptive coping strategies lead to criminal behavior) (23).

According to the authors of the GLM, the following types of problems can occur while people are pursuing life goals: *internal and external capacity* (or obstacles), *scope*, *means*, and *coherence* (24). *Internal capacity* refers to cognitive, psychological, and behavioral abilities, whereas *external capacity* means the availability of support or employment, for example. These capacities represent empirically identified criminogenic needs (25), so they can be seen as dynamic risk factors and treatment goals. A lack of *scope*, i.e., neglecting important goods, which is often associated with capacity problems, results in failure to achieve primary goods and results, for example, in physiological dysfunction, psychological distress that leads to mental health problems, or social maladjustment (24). A problem with *means* occurs when a primary good is achieved in a dysfunctional manner that decreases the probability of goal attainment. *Coherence* expresses the interdependence between an individual’s goals: Horizontal coherence is defined as the mutual relationship of goods in a consistent and enabling way, whereas vertical coherence describes the individual hierarchical ranking among goods.

### Recovery model

During the course of the recovery movement, in 2003, Repper and Perkins (26) proposed a new model of clinical practice for mental services that aims at improving the quality and meaning of service users’ lives by abandoning the paradigm of the necessary absence of symptoms. The primary focus of the model is personal recovery, understood as the achievement of skills necessary to maintain or restructure meaning and value in one’s existence. The model, unlike the two analyzed above, was proposed for general psychiatric practice as an effective measure to overcome any symptoms and deficits remaining despite therapy during the treatment of mental illness (27). It is often defined as “[…] a deeply personal, unique process of changing one’s attitudes, values, feelings, goals, skills and/or roles […] and a way of living a satisfying, hopeful and contributing life even with the limitations caused by illness” (8).

In the first decade of the 2000s, several Western forensic mental health (FMH) services began a paradigm shift from so-called forensic rehabilitation (28) to the concept of personal recovery. Their goal was to move from models and measures focused on the risk of recidivism to a framework more oriented toward the concepts of clinical, functional, and personal recovery and achieving improvement in both clinical and social functioning. Often, this shift involves blended, institution-specific forms of rehabilitative treatments that contain elements of both personal recovery and forensic rehabilitation, highlighting the changes occurring within the forensic setting.

One of the best-known conceptual frameworks to synthesize the personal recovery-based model is the CHIME framework proposed by Leamy et al. (29), which was originally established in general psychiatry. The acronym synthesizes the five basic elements of the recovery process: (1) connectedness, (2) hope/optimism, (3) identity, (4) meaning in life, and (5) empowerment (29).

Within the FMH setting, *connectedness* is reinterpreted as the possibility of developing a healthy and positive interaction with members of the nursing staff and other individuals on the ward, and trust in caregivers is a pivotal element that must be achieved and cultivated during hospitalization in order to achieve the desired outcomes. Criticisms of the elements of *connectedness* are that FMH settings offer limited opportunities for interaction and that the most popular ways of implementing the model contain no reference to connecting with the external environment.

*Hope and optimism* about the future must be achieved by reframing past experiences and helping individuals to imagine themselves in a life after discharge in which they are free of all the elements that characterized their experience. From time to time, individuals have difficulty visualizing a concrete end to the inpatient period and being free from the burden of actions committed or perceptions of their external environment. This point is perhaps of the greatest interest in the forensic population because the stigma is twofold: Individuals have to face the implications not only of their underlying diagnosis but also their conviction.

The third point concerns *identity*, i.e., redefining one’s self as a result of the crimes committed and the trauma suffered and constructing a new identity that is healthier for life during treatment and after discharge. However, the tools available to individuals in facilities appear to be severely limited, and it is difficult for many to finish this work during their time in FMH facilities.

*Meaning in life* is defined as building a new set of goals and motives for making the most of one’s experience, with a focus on life after discharge. During inpatient treatment, it is interpreted as meaningfully using the time available for one’s recovery. The problems that individuals most often encounter in the inpatient period are the absence of deep meaning (i.e., a lack of QoL) and the inability to find sufficient resources to begin living their experience in a meaningful way (boredom).

*Empowerment* is perhaps the most critical point within the forensic psychiatric setting. Providing tools to increase patients’ knowledge about their illness, possible treatments, and legal procedures is certainly a starting point, but the ultimate goals concern autonomy and independence after discharge. However, health professionals must keep in mind that FMH facilities are a highly disempowering setting because many of the activities that should be inherent in personal autonomy are delegated to staff or regulated by standardized procedures and cannot be directly experienced by patients.

The specific characteristics of the population of users of FMH services naturally impose limits and questions about the acceptability and usefulness of this framework in people who may represent a risk to themselves and others. The autonomy of the individual, which is a fundamental characteristic of successful personal recovery, is (or is understood to be) limited in FMH services. Several attempts have been made to repurpose such frameworks for the specific characteristics of FMH, such as the one recently proposed by Senneseth et al. (30), which will be described in the next section.

## Application and evidence for the treatment of mentally disordered offenders

### Evidence for rehabilitation with the risk-needs-responsivity model and good lives model

As a rehabilitation model, the RNR model focuses on risk management. Its efficacy in reducing the risk of re-offending has been empirically demonstrated over the course of 30 years (31). Studies on the RNR model often addressed its main principles and the related central framework of eight risks/needs. For example, a recent book chapter reviewed the current empirical status of these main principles (31). By reviewing 14 meta-analyses, the authors found that the principle of *risk* had mixed results, the principle of *need* was well supported, and the principle of *responsivity* was supported but rarely studied. However, only one of these meta-analyses explicitly addressed the use of the RNR model in MDOs and found support for the principles of both *risk* and *need*. In particular, this meta-analysis of 126 studies on MDOs found good support for the central eight risk/need factors (32). The factors predicted general recidivism with small to moderate effect sizes (the strongest predictors were past and current substance abuse, procriminal attitudes, and an antisocial personality pattern) and violent recidivism also with small to moderate effect sizes (the strongest predictors were an antisocial personality pattern and criminal history).

According to another review article, the empirical validity of the RNR model may arise from the fact that the model is a multifactorial rehabilitation theory that is “global, […] broad in focus and lacking sufficient detail to directly shape the design of specific interventions” (33). The authors of the article argue that this fact results in strengths, such as empirically well-supported basic principles; a valid explanation of pre-existing research findings on recidivism; and practical utility. On the other hand, they write that it also implies some weaknesses: The theoretically underdeveloped responsivity principle serves as “a catch-all category” that fails to explain why something works or does not work with some offenders. The central eight risk/need domains may be empirically well supported, but the RNR model does not explain how these domains are related to each other and does not provide the mechanisms to change them for different offenders (33).

The GLM is often considered as an alternative rehabilitation model that incorporates ideas directly related to the deficits of the RNR model. An article by prominent supporters of the GLM describes how the model may work in forensic psychiatry (34). In the authors’ view, traditional approaches in forensic psychiatry often subjectively mix risk/need principles with psychopathology models, which may result in an unsystematic and fragmented treatment approach. In contrast, the GLM—as an overarching rehabilitation theory—enables the integration of personal goals, risk reduction, and psychiatric treatment while also addressing ethical issues such as human rights. The authors claim that MDOs may have limited motivation to participate in rehabilitation programs that focus on avoidance goals and are not linked to personal values and aspirations. By using a case example of a patient with schizophrenia, the authors describe how mental disorders hinder the drive for a good life and how using GLM principles can be used to address patients’ needs.

In contrast, other authors state that differences between the GLM and RNR model are rather semantic (35). They discuss similarities and differences between the two models and conclude that the RNR model is effective in reducing general, violent, and sexual recidivism and is also effective in female offenders. In their opinion, some of the primary goods of the GLM are inverse versions of the central eight risk/need domains of the RNR model, for example the *excellence in play* and *excellence in work* in the GLM are related to *problematic circumstances at school/work* and *few/no prosocial recreational activities* in the RNR model. However, the authors suggest that the RNR model might be improved by considering research about therapeutic relationships and issues of mental health and mental disorders, and they propose using an adapted version of the RNR model, the integrated risk-need-responsivity model, which incorporates empirical findings from sex offender research.

The GLM provides a more goal-oriented and strengths-based offender rehabilitation than interventions based on the RNR model. A recent systematic review of 17 articles provides some insights into the empirical evidence for the main assumptions of the GLM and outputs for offender rehabilitation (36). The authors found mixed results regarding the main assumptions of the GLM. One assumption is that offending is an attempt to fulfill universal primary goods that the person is searching for. Some qualitative studies suggest that offending is related to some degree to this search: Studies with mentally disordered participants showed that primary goods are related more to psychopathology than to recidivism; however, psychopathology was found to be related to recidivism, suggesting an indirect path from the search for primary goods to offending. A 3-year follow-up study found a moderate relationship between unmet needs and recidivism, but the relationship disappeared when the offenders’ risk profile was considered. However, violent recidivism in high-risk offenders was found to be three times less likely when they were satisfied with their life. Regarding the outcomes of offender rehabilitation with the GLM, the reviewer found comparable attrition rates for the GLM and interventions based on risk/need. Findings on psychometric measures such as self-esteem, empathy, and change motivation showed that GLM-based interventions were at least as good as those based on risk/need. In addition, service users were found to have a generally positive opinion of the GLM. However, one study reported concerns regarding the balance between promoting goods and reducing risks in GLM-based interventions.

In general, opponents of the GLM often criticize that the model lacks clear evidence proving that it reduces re-offending. The GLM theorizes a direct and an indirect path from QoL to offending and that QoL can be addressed by GLM-based interventions. A longitudinal study investigated these assumptions by measuring QoL within the first 3 weeks of imprisonment in a sample of detained female adolescents (37). In a follow-up measure, reincarceration status, mental health problems, and offending were recorded 6 months after discharge. In support of the GLM, by using a structural equation model the authors found a significant indirect path from QoL *via* mental health problems to offending; however, they found no support for a direct path from QoL to offending. In a recent review of six studies, Zeccola et al. evaluated whether GLM-based interventions reduce recidivism in offenders (38). Four studies investigated the efficacy of the GLM in sex offenders; one study, in mentally disordered sex offenders (MDSOs); and one study, in violent offenders. Overall, the six studies had methodological problems and, consequently, an increased likelihood of bias. No study provided effect sizes. Nevertheless, the studies showed that the GLM may reduce recidivism and dynamic risks in sex offenders. However, in GLM-based treatment the length of treatment must match the needs of offenders. The study that investigated GLM in MDSOs could not report mean outcomes because of incomplete questionnaires. The authors concluded that current research is unable to confirm that GLM-based interventions show efficacy in preventing recidivism.

### Evidence for the use of the recovery model in forensic psychiatry

Despite several attempts to demonstrate the efficacy of the RM, one main problem remains: There is a lack of consensus on the exact definition of recovery and how to measure it. Although the last decade has seen a growing interest in the RM in protected settings, the literature on this topic remains sparse. One study used the Recovery Journey Questionnaire to evaluate the recovery approach in a medium secure psychiatric unit (39). The questionnaire has been proposed as a method to standardize the measurement of service users’ experiences regarding their personal recovery. The authors found that the Recovery Journey Questionnaire correlated positively with QoL measures and negatively with the hopelessness scale. Treatment engagement was identified as one of the most important factors in determining the effectiveness of the RM. Furthermore, the Recovery Journey Questionnaire was able to predict treatment motivation, treatment engagement, and social problems independent of measured QoL. The results of this study highlight the possible effects of personal recovery in secure settings.

Another good example of the usefulness of the RM is a review of the literature on recovery and the GLM that discusses the applicability of these concepts in MDSOs (40). The author found a convergence between GLM and secure recovery because both are client centric and related to positive psychological ideas such as enhancing skills, building social capital, and developing valued social roles. They noted that MDSOs and other sexual offenders have similar criminogenic needs that require “careful risk assessment […] regardless of positive changes in the mental disorder” (41). Regarding empirical evidence for secure recovery and the GLM, the author concludes that “[while] there is growing evidence for the clinical utility and face validity of secure recovery and GLM applied to MDSOs, more research, particularly on engagement, recidivism, sustained community living and other behavioral outcomes, is required” (40).

In secure settings, the RM has been often investigated in qualitative studies. A recent systematic review included 19 such studies and two former systematic reviews on forensic mental health service users’ perspectives on recovery (30). The authors found support for the five recovery processes of the above-mentioned CHIME framework. However, they added a sixth process, which they called *safety and security*, i.e., the need to feel “protected from hostile people and environments and the active practice of self-management of risk” (30). In addition, they identified challenges and barriers to personal recovery in forensic mental health services. To name a few, the recovery process of connectedness was challenged by feeling disconnected from social networks, having sparse social support, and feeling lonely. Hope and optimism were affected by “uncertainty about future discharge or length of stay,” for example (30). Service users felt disempowered because of negative attitudes of staff toward them and “rules and restrictions perceived as punitive and pointless” (30). The authors conclude that they “cannot identify any obvious conflict between forensic recovery-oriented practice and the system’s security needs” (30).

Some researchers have studied the relationship between personal recovery and the recovery pathway (i.e., individuals’ moves from higher to lower levels of security and to discharge) in forensic psychiatry (42, 43). On the basis of their findings, they conclude that the recovery pathway shows “a clear and understandable connection between risk management and care planning, thereby providing patients clarity and hope when working toward their own recovery” (42). A prominent measure in this area is the Dangerousness, Understanding, Recovery and Urgency (DUNDRUM) quartet, which was developed by researchers and forensic psychiatrists at the National Forensic Mental Health Service of Ireland and the Academic Department of Psychiatry at the University of Dublin (44). The quartet consists of four structured professional judgment instruments that are used to assess admission triage, urgency, treatment completion, and recovery.

The authors of one research article hypothesized that patient-rated treatment completion and recovery scores predict levels of security and conditional discharge with similar accuracy as staff-rated scores on the same scales (42). In addition, they assumed that the concordance between patient-rated and staff-rated scores predicts conditional discharge. Although their study found an association between patient- and staff-rated scores, patient-rated scores were more optimistic. Furthermore, patient ratings did not predict moves between levels of security or conditional discharge; however, concordance between patient and staff ratings did predict both conditional discharge and negative moves between levels of security. The authors concluded that “[those] who progressed to conditional discharge were those with the lowest (best) scores on the DUNDRUM-3 programme completion and DUNDRUM-4 recovery scales, and also the least differences between clinician ratings and self-ratings (42).”

A 13-month prospective study in a high security forensic psychiatry assessed patients’ risk of violence (with the Historical, Clinical and Risk Management-20 [HCR-20]), program completion and recovery (with the DUNDRUM quartet), and overall functioning (45). The goal of the study was to evaluate whether risk, need, and recovery correspond to placements on lower or higher security wards within a high secure hospital and to moves to a medium secure hospital. The authors found that current, future, and dynamic risk scores and overall functioning predicted placements and moves but that past risk scores did not. Better scores on the program completion and recovery scales corresponded to placements in lower security wards and moves to the less secure facility. The authors concluded that the results demonstrate the practical use of all the scales as real-world outcome measures that correspond to clinical decisions.

## Comparison of the main rehabilitation principles

### Role of individual protective factors and personal resources

Individual protective factors and personal resources—or more precisely, the lack of them—are addressed by the RNR model principles of need and responsivity. Practitioners are advised to provide interventions that first address the criminogenic needs of the individual and second are in line with the individual’s personal characteristics, which may interfere with the efficacy of the chosen intervention (31). RNR places the focus on *risk*, not only because treatment is recommended for those with high-risk profiles, but also because individual case formulation consists of a composition of risk factors, and resources are mainly defined as the absence of deficits. Of the *Big Eight* criminogenic factors, more than half are associated with antisocial behavior or cognitions. These factors might be relatively easy to address with treatment if they can be seen as symptoms of a severe mental disorder. However, the model provides only limited information on how the different principles interact and whether they might follow a different hierarchical order when applied in MDOs.

In contrast to the RNR model, the GLM recommends not merely addressing deficits of personal protective factors and personal resources as criminogenic needs but also providing the individual with an alternative strengths- and goals-based concept for living their life. Rooted in positive psychology, GLM emphasizes the role of offending behavior in an individual’s search for a good life. Thus, practitioners are advised to discover, together with the individual, the individual’s personal goals and related personal strengths, an approach that results in “[motivating] them to live better lives and [to equip] offenders with the capabilities and resources to obtain primary goods in socially acceptable ways” (46).

The term “personal recovery” itself highlights the importance of using personal protective factors and personal resources on the path to recovery. The recovery processes suggested by the CHIME framework can be seen as shared issues of users that may function as personal resources for their own recovery path. The adaptions for FMH services have already taken into account that safety and security are additional shared issues in forensic psychiatry that users view as a necessary base for their recovery process (30, 47). The five recovery principles and their operationalization are formulated positively and cover the main aspects of the issues that MDOs have to deal with during treatment.

### Situational and environmental factors of rehabilitation and reoffending

The central eight risk/need factors of the RNR model include some important situational and environmental topics that can be focused on by practitioners, namely education and employment, leisure/recreation, family and marital relationships, and antisocial peers. Because these factors are directly related to offending, at least empirically, addressing them with well-chosen interventions is thought to lead to a reduction of reoffending (31).

As stated above, some of the 11 primary goods of the GLM are worded as reverse versions of the risk/need factors of the RNR model, and addressing them may reduce reoffending (35). Because the GLM views offending as either a direct search for primary goods by socially unacceptable means (direct pathway) or an accumulation of negative experiences that eventually lead to a loss of control of the situation and criminal activities (indirect pathway), the impact of situational and environmental factors is highly important within the GLM (37). Therefore, improving one’s QoL, setting positive life goals, and working on one’s strengths is thought to “block” both the direct and indirect pathways of offending.

As stated at the end of the previous section, ensuring safety and security are important processes of recovery shared by forensic patients (30, 47). Furthermore, guaranteeing safety and security for the public is a major requirement for conditional discharge from forensic psychiatry. Thus, personal recovery, or more precisely secure recovery, and the prevention of reoffending might be seen as being related to each other (41, 42).

### Participation of the patient in the assessment procedure, treatment planning, and evaluation

One of the main criticisms of the RNR model is that it does not consider the patient’s perspective, such as their core interests. For example, a critical response article by GLM supporters claimed that the only reason why practitioners of the RNR model build “a working alliance with offenders is to empower therapists rather than to establish a relationship through which offenders can embark on life changes in a safe and collaborative way” (48). Supporters of the RNR model argue that the RNR model provides additional principles that recommend assessing offenders’ strengths and non-criminogenic needs, for example. However, they admit that such principles have been “the most overlooked concept in the RNR model” and have “been widely ignored, and sometimes actively discouraged” and that “many proponents of the RNR model have focused exclusively on risk and needs” (31).

Because the GLM aims to improve individuals’ resources to enable them to live a good life, participation of the individual is crucial. For example, a published GLM tool kit for MDOs provides five tools to guide GLM-based interventions through assessment, rehabilitation planning, and execution while considering the goals and strengths of the MDO (46). To understand the concepts of GLM and to benefit from its rationale, an offender must have relatively good cognitive resources and a high ability for self-reflection. Consequently, on an individual level, the model’s success might depend on the degree to which a person can identify with these rather abstract concepts and initiate behavioral changes.

As a user-oriented model, the RM emphasizes patients’ perspective of their own treatment. Recovery is seen as a highly individual concept. Thus, practitioners are advised to help patients along their recovery path, which aims to achieve a sense of purpose, QoL, and mastery in life (30). Patient participation is therefore a mandatory requirement to achieve the treatment goals of the RM.

### Outcome criteria

The authors of the RNR model focused on general criminal risks and deficits to address the issue of psychological interventions that were often conducted in offenders and were ineffective in preventing re-offending (31). The RNR principles capture the empirical observation that “our best interventions, when applied to our most selectively chosen offenders, were capable of reducing the recidivism by up to 50%, while ‘inappropriate’ treatment interventions that failed to follow these principles had a slightly deleterious effect on offender outcome” (31). This observation is the reason why the RNR model mainly focuses on reducing criminological outcome criteria and providing a structured assessment of recidivism with validated instruments such as the HCR-20. Meanwhile, the assessment of non-criminogenic outcome criteria, such as improving QoL or mental health, is also recommended (31).

The GLM aims to reduce recidivism by promoting offenders’ personal goals (34). Research has also investigated the relationship between the GLM and change motivation and psychometric aspects, such as self-esteem (36). Intuitively, QoL appears to be a consequential outcome variable of the GLM. However, previous studies used QoL as an independent variable in the assessment of re-offending, for example (36, 37). Further clarification of the relationship between QoL and “good lives” (GLM) was also requested in a recent critical review on strengths-based approaches in various disciplines (49).

Recovery itself is the goal and therefore the intended outcome of the RM. In addition, research on personal recovery describes important recovery processes, such as those suggested by the CHIME framework, rather than measuring outcome criteria (50). This approach makes it difficult to clearly define additional outcome criteria. However, a relationship between recovery and QoL has been both theoretically assumed and empirically demonstrated (39). In addition, as mentioned earlier, some promising research indicates a relationship between personal recovery and the recovery pathway in forensic psychiatry.

## Discussion

The main goal of forensic psychiatric treatment is to decrease the risk of re-offending. Thus, a core issue regarding the applicability of strengths-based approaches in the treatment of MDOs is the role of personal development and personal agency in criminal behavior. One of the roots of the GLM is the self-determination theory of motivation, which states that individuals seek growth and positive development and that both of these can be found in social environments that support the satisfaction of three basic psychological needs, namely a need for competence, need for autonomy, and need for relatedness (43). This humanistic view assumes that criminal behavior results from a dysfunctional striving for personal development and personal agency, an issue that can be well addressed in treatment. As regards aspects of self-determination, one must consider that most severe mental disorders have an impact on mental capacity and decision-making processes, at least temporarily (51–53). If symptoms affect executive functioning in particular, planning skills and goal-orientated behavior may be reduced. Although the GLM takes this into account with the criterion *internal capacity*, its “ideal form” might exclude a significant proportion of MDOs.

In our opinion, another critical aspect of strengths-based approaches such as the GLM is that they may overestimate the role of personal development and personal agency in criminal behavior. As put forward by Hirschi in the influential book “Causes of Delinquency” (54), an important question is why most people *don’t* engage in criminal behavior. This question implies that certain protective/control processes exist that normally ensure socially acceptable behavior (55, 56). A fulfilling life that includes desirable opportunities for personal development and personal agency may increase the probability of such behavior and may be the single protective factor that should be addressed in the treatment of MDOs. However, having or not having a fulfilling life is insufficient as an explanation for criminal behavior and as an overarching rehabilitation paradigm for forensic psychiatry. The actual problem is that multiple protective processes may be disrupted or less effective in high-risk offenders, especially in MDOs with severe mental disorders. Proponents of the GLM claim that risk-need approaches are too pessimistic and deficit focused (57). However, in the same manner, one could simply claim that the humanistic view may be too optimistic and growth focused.

A recent review on strengths-based approaches used by professionals in various disciplines who work with MDOs or rather, in the authors’ preferred terminology, “patients with mental disorders,” puts forward similar arguments (49). First, the authors note that strengths-based approaches share key ingredients that relate to individual and interpersonal competencies and community resources, and they agree with having a positive view of humankind, in particular with the assumption that clients have capacities to grow. Then, they problematize that within fields that work with patients with mental disorders, these shared views may lead to dilemmas and challenges. For example, emphasizing self-determination may lead to an underestimation of the influence of contextual factors and society on individual criminal behavior. Another issue is that strengths-based approaches need to more clearly formulate the relationship between strengths/protective factors and dynamic risk factors. In this regard, the authors promote the holistic perspective of strengths-based approaches. Most importantly, they argue that “living apart together” is important for the various disciplines (49). Such an approach has been requested not only for different strengths-based approaches, but also for risk-oriented models and mental health perspectives. We agree with the authors’ conclusion that different approaches and paradigms should be seen as complementing rather than competing with each other (49).

In this regard, we want to take a closer look at the theoretical underpinnings of the RNR model. The risk-need orientation is grounded in social learning theories, in particular the GPCSL. The model assumes that criminal behavior is learned by positive enforcement and is likely to occur when individuals expect that “the rewards and costs for crime outweigh the rewards and costs for prosocial behavior” (58). To explain criminal behavior, it emphasizes social learning from environmental factors that reward criminal behavior, such as having procriminal companions, rather than from individual/motivational factors, such as personal agency. Criminal risks/need factors provide social learning opportunities that result in procriminal attitudes and cognitions, which then increase the occurrence of criminal behavior in criminally rewarding situations. This point of view is pessimistic in that it assumes that re-offending is likely to occur when criminal risks remain because the needs maintain the positive enforcement of criminal behavior.

The GPCSL theory has weaknesses, such as an outdated and oversimplified assumption about criminal decision-making. As discussed in a recent annual review on self-control and crime, criminal behavior does not occur because of a simple, rationale calculation of rewards and costs but is related to multiple dynamic processes, such as perceiving stimuli as a temptation for criminal behavior and having dysfunctional impulse control (55). However, the GPCSL correctly recognizes that ongoing exposure to certain criminal risks strongly predicts the probability of re-offending. The authors of the RNR model referred to these risks as *criminogenic* needs. Focusing on them in the treatment of MDOs is as appropriate today as it was 30 years ago, when the RNR model was established. However, treatment must not be based solely on risk management while neglecting strengths and personal development. In contrast to the GLM, we also question focusing “on promoting offenders’ personal goals while at the same time reducing their risk for future offending” (57). Instead, we advise incorporating strengths- and recovery-based approaches beyond crime prevention, in particular by focusing on addressing MDOs’ criminogenic needs while at the same time considering their personal goals and search for personal recovery.

The authors of a recent article made a valuable attempt at incorporating strengths- and recovery-based approaches in forensic psychiatry beyond crime prevention (12). They deal with the applicability of strengths- and recovery-based approaches in forensic psychiatric in the UK and believe that forensic psychiatry is based on the idea that the same setting can both provide mental health care and reduce the risk of re-offending in MDOs. The authors add that this idea is related to meeting psychiatric and legal standards such as security and social control. Such legal standards would challenge the full realization of strengths- and recovery-based approaches, which require degrees of freedom for service users. However, the authors claim that Article 8 of the European Convention of Human Rights and Fundamental Freedoms offers a legal option for strengths- and recovery-based approaches because it emphasizes individual rights, such as the pursuit of personal development, while considering public interests, such as safety and the prevention of crime. Thus, in our opinion, engaging in secure recovery that includes in equal measure both measurable risk management and working on a meaningful life—notwithstanding the limitations of the psychiatric disorder—may be a moderate and practical rehabilitation goal to guide service users and practitioners toward conditional discharge.

Another important difference between MDOs and general offender populations that must be considered when applying models from general offender treatment to MDO treatment is the link between criminal responsibility and free will or autonomous decision-making. Although it is reasonable to assume that someone with an antisocial personality disorder willingly decides to offend, this is not the case in a patient with an acute psychotic episode, which is the basic reason why the legislation in many countries explicitly considers individuals with severe psychological problems as having diminished or no criminal responsibility (59). Consequently, the RNR model and the GLM, both of which understand criminal behavior as more or less intentional acts, do not cover this type of offending, and one can question what additional effects might derive from the application of such models if the main risk factor for reoffending has been identified as the mental disorder itself. The RM does not have this kind of conceptual gap because it originates from the treatment of mental disorders. However, it can also be criticized as being slightly too optimistic and assuming a prototypical ideal course of severe mental illness in which individuals reach complete or at least a very high extent of remission. Unfortunately, these ideal treatment outcomes are rare in forensic psychiatric settings, where a large proportion of patients have a chronic illness and may not experience full remission.

Central aspects of using the discussed models for the rehabilitation of MDOs have to be critically discussed from a methodological and ethical perspective. The first consideration that arises from analyzing the different models presented here is that none of them was initially designed and intended for FMH services and their users. In fact, the models were initially developed either as rehabilitation and recidivism risk-reduction programs in offenders without severe mental pathology (the GLM and RNR model) or for individuals with a psychiatric disorder in voluntary care (RM). The models have since been repurposed and applied in FMH services, which represent a specific overlap between the two environments in which the models were developed (31, 34, 39). However, the question is whether the model processes can sufficiently address the specific therapeutic and rehabilitative needs of a population that is different from the source population.

In this review, we analyzed several efforts to appropriately repurpose the models to the FMH services setting and demonstrate their effectiveness. The first ethical and methodological problem is that this process is the psychotherapeutic equivalent of the off-label use or secondary indication of a drug originally developed for a different condition. It is by no means certain that such models will be able to cover all the therapeutic, rehabilitative, and precautionary needs of FMH services. In a retrospective analysis, this approach is synonymous with a lack of specific effort to create a model designed to accommodate the forensic characteristics of the individuals in question. There may be two explanations for this lack of effort: The first is the ongoing lack of clarity about the classification of forensic psychiatry and which discipline has more responsibility. Indeed, FMH is often perceived as being detached from general psychiatry, both physically and in terms of the service users and the specific training of the staff working there. At the same time, FMH can be considered as belonging to the field of justice and criminal rehabilitation, although the specific needs of the users and the strong medicalized character make it appear more akin to psychiatry. This basic ambiguity makes it difficult to identify which area is of greatest interest in initiating reform and research processes in the field (60). The second explanation is the limited literature available, which may be due to the difficulties in obtaining ethics committee approval for studies in this population and in generalizing results (because of the usually small sample sizes and the small amount of published studies that offer data sharing) (61–63).

The articles presented in this study reveal several critical elements concerning the application of the three models in the FMH setting. These elements are extremely specific to the characteristics of the users and staff and of the setting itself. At the ethical level, the difficulty of implementing these models (with the associated training required for staff) and, in the absence of clear national and international guidelines, the consequent fracturing of the organization of individual FMH centers, which very often reaches the regional level, represents a lack of consensus that would be unacceptable in other areas of medicine today. The scientific community is aware of the problem, but they need to make a greater effort to amplify the capacity for specific research and the creation and implementation of projects dedicated to FMH. More discussions at an international level to identify a direction for both research and user assistance would be highly desirable. It should be noted that we couldn’t find any randomized-controlled trial which tested the outcome of RNR, GLM, or RM. This type of study is indeed difficult to conduct considering FMHs ethical, legal and psychiatric circumstances. Nevertheless, we want to encourage researchers to conduct well-designed outcome studies as they would be a welcome contribution to improve treatment in FMH.

## Author contributions

IF, DZ, and ML conceptualized the review and wrote the initial draft of the manuscript. ML and DZ conducted the main part of the literature search. MD and MF contributed to the outline of the review, the introduction, and the discussion section. All authors read and approved the final version of the manuscript.
